# Fatal Outcome Due to Kounis Syndrome Following Fluorescein Retinal Angiography: A Case Report

**DOI:** 10.3390/diagnostics14111092

**Published:** 2024-05-24

**Authors:** Vincenzo Cianci, Claudia Pitrone, Daniela Sapienza, Alessandro Meduri, Antonio Ieni, Patrizia Gualniera, Alessio Asmundo, Cristina Mondello

**Affiliations:** 1 Department of Biomedical and Dental Sciences and Morphofunctional Imaging, Section of Legal Medicine, University of Messina, Via Consolare Valeria, 1, 98125 Messina, Italy; clapitrone@gmail.com (C.P.); daniela.sapienza@unime.it (D.S.); patrizia.gualniera@unime.it (P.G.); mondelloc@unime.it (C.M.); 2 Department of Biomedical and Dental Sciences and Morphofunctional Imaging, Section of Ophthalmology, University of Messina, Via Consolare Valeria, 1, 98125 Messina, Italy; alessandro.meduri@unime.it; 3 Department of Human Pathology of Adult and Childhood “Gaetano Barresi”, University of Messina, Via Consolare Valeria, 1, 98125 Messina, Italy; antonio.ieni@unime.it

**Keywords:** Kounis syndrome, anaphylaxis, myocardial ischemia, forensic pathology, post mortem biochemistry, post mortem immunohistochemistry, retinal fluoroangiography

## Abstract

Kounis Syndrome (KS) is a clinical entity triggered by allergic or hypersensitivity reactions capable of inducing acute coronary events. Several causes can induce KS, including drugs and insect stings. Here, a rare case of post mortem assessment of fatal KS related to fluorescein retinal angiography has been reported. An 80-year-old man in follow-up for a retinal vein thrombosis underwent a retinal fluoroangiography. Approximately 30 min later, the patient complained of sweating and dizziness, and suddenly lost consciousness due to a cardiac arrest. Despite the immediate cardiopulmonary resuscitation, he died. The autopsy revealed foamy yellowish edema in the respiratory tract and coronary atherosclerosis with eccentric plaques partially obstructing the lumen. The routine histology highlighted lung emphysema and myocyte break-up with foci of contraction band necrosis at the myocardial tissue. Biochemistry showed increased serum tryptase, troponin, and p-BNP. Activated and degranulated (tryptase) mast cells were detected, using immunohistochemistry, in the larynx, lungs, spleen, and heart. Acute myocardial ischemia due to allergic coronary vasospasm related to fluorescein hypersensitivity has been assessed as cause of death. KS-related deaths are considered rare events, and the post mortem assessment of KS quite difficult. The integration of several investigations (gross and microscopic examination, biochemistry, immunohistochemistry) can provide useful findings to support the diagnosis, helping to reduce the unrecognized cases as much as possible.

## 1. Introduction

Anaphylaxis is a life-threatening reaction characterized by an acute onset of symptoms involving different organs and systems which require an immediate medical intervention [1]. The rate of fatal anaphylaxis is low but, in recent years, an increased frequency of hospitalization for food- and drug-induced anaphylaxis has been reported [2]. The signs and symptoms of anaphylaxis are highly variable; those involving skin and mucosae are reported in >90% of cases, followed by signs/symptoms involving the respiratory and cardiovascular systems (>50%) [3]. The most common elicitors of the hypersensitivity reaction are represented by food, drugs, and Hymenoptera venom [4].

Among the spectrum of allergic reactions, Kounis syndrome (KS) is defined as an acute coronary syndrome related to the release of allergic inflammatory mediators which can affect the coronary arteries [5]. It is considered a complex multisystem disease including any coronary syndrome related to mast-cell-associated disorders and inflammatory cell interactions [6].

In general, the post mortem assessment of an anaphylactic death is considered a challenge because frequently no pathognomonic gross and microscopic findings are detected at autopsy. Thus, the diagnosis must be performed by the integration of several findings emerging from different types of investigations, among which biochemistry and immunohistochemistry are considered the most useful [7]. This post mortem approach is crucial also in cases with suspicion of KS, in which particular attention should also be given to the heart and coronary arteries [8].

Here, the authors report a rare fatal case of Kounis syndrome due to retinal fluorescein angiography, highlighting the main forensic investigations to apply in such cases and the contribution of each one in reaching a correct post mortem diagnosis.

To the best of the authors’ knowledge, there are no other cases reported in the literature documenting a death due to Kounis syndrome following retinal fluorescein angiography.

## 2. Case Report

An 80-year-old man was in follow-up for a central retinal vein thrombosis of the right eye. For this reason, he underwent a retinal fluoroangiography and, at the end of the investigation, a transitory and largely unexplained illness occurred. The patient then continued the follow-up planned for the aforementioned pathology. After one year, the man underwent a second retinal fluoroangiography through the intravenous injection of 5 mL of sodium fluorescein (20% solution). Approximately 30 min later, the patient complained of sweating and dizziness, and a few minutes later he suddenly lost consciousness and a cardiac arrest occurred. Despite the rapid intervention of the healthcare practitioners, the subject died, as the practiced resuscitation measures proved ineffective.

The autopsy revealed no signs of edema at the tongue and the larynx; both poor dense yellowish mucus on the larynx and bronchial mucosa and foamy yellowish edema leaked from the bronchi were observed. The heart (weight 445 g, transverse diameter 12 cm, longitudinal diameter 11 cm) revealed coronary atherosclerosis with eccentric plaques, partially obstructing the lumen, especially in the descending anterior coronary; the wall thicknesses were 18–20 mm at left ventricle, 4–5 mm at the right ventricle, and 20 mm at the interventricular septum; and there was modest sclerosis of mitral and aortic valve leaflets.

**Figure 1:**
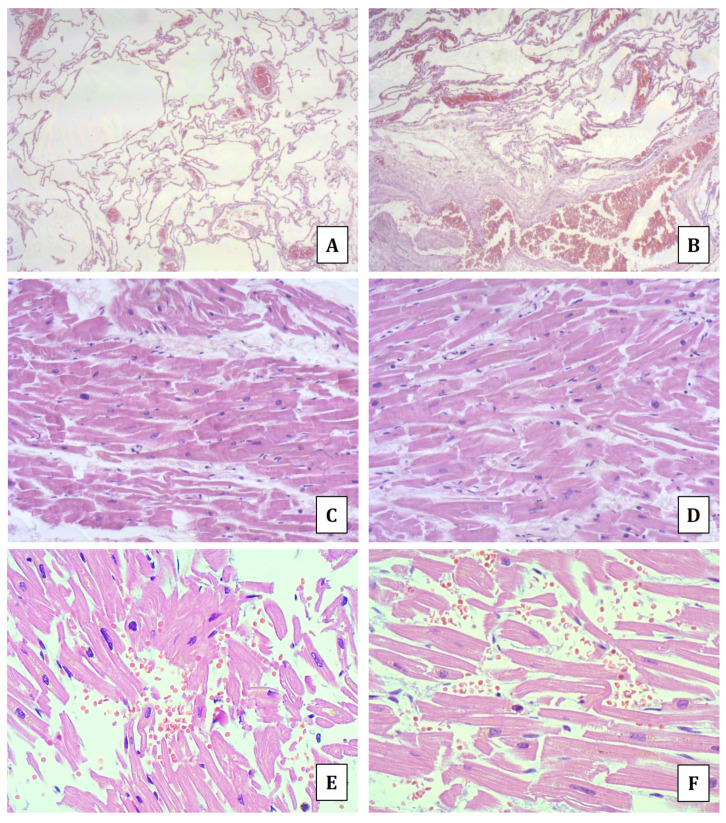
Hematoxylin and eosin staining. Lung tissue showing alveolar septa rupture with large areas of emphysema (**A**) and bronchial/intra-alveolar hemorrhagic edema (**B**). Heart tissue with myocyte break-up and foci of contraction band necrosis (**C**,**D**), and myofiber breakage and disarrangement with microhemorrhages (**E**,**F**).

At routine histology, the larynx had normal architecture, while the lungs showed alveolar septa rupture with spread and large areas of emphysema and a few scattered lympho-monocytic infiltrates (Figure 1A); there were also scattered bronchial and intra-alveolar hemorrhagic edema (Figure 1B). The main myocardial tissue findings were spread myocyte break-up with hypercontraction of myofibers and scattered foci of contraction band necrosis (Figure 1C,D). There were also scattered foci of both disarranged myocytes and wavy fibers (Figure 1E,F); spread areas of fibrosis were also detected, as well as notes of partially and eccentrically stenosing atheromasia of the coronary branches due to fibrosclerotic thickening.

**Figure 2:**
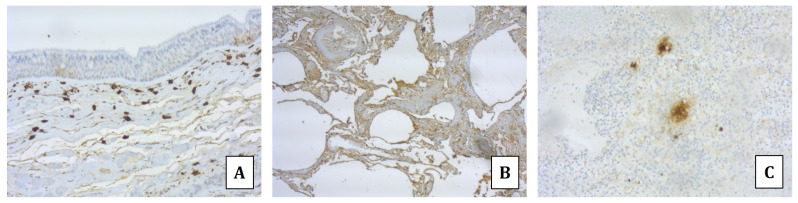
Immunohistochemistry by anti-tryptase antibody. Tracheal tissue: several non-degranulated mast cells and few degranulated cells together with scattered immunopositivity in epithelial and sub-epithelial layers (**A**). Lungs: scattered immunopositivity in both perivascular interstitium and interalveolar septa (**B**). Spleen: immunopositive mast cells surrounded by a positive halo, due to the tryptase degranulation (**C**).

**Figure 3:**
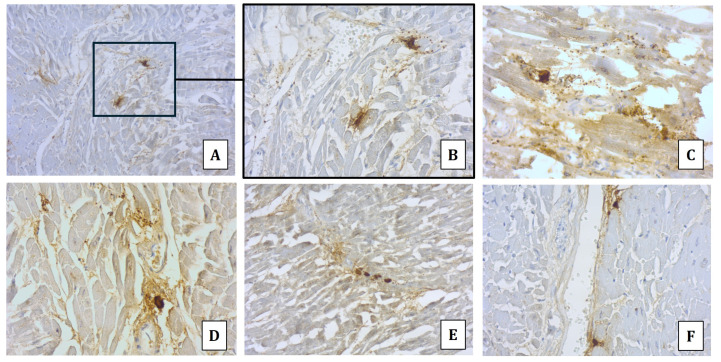
Immunohistochemistry by anti-tryptase antibody of myocardial tissue: immunopositive mast cells surrounded by both a positive halo and spread degranulation, mainly localized at interstitial space (**A**–**E**) and perivascular regions (**F**).

Immunohistochemical analyses using monoclonal mouse anti-tryptase antibody (Roche Diagnostics, code 760-4276) on trachea, lung, spleen, and heart specimens were also performed. The analyses revealed an intense antibody expression in the tracheal tissue, characterized by an immunopositivity for mast cells, with some of these surrounded by a positive halo (degranulated tryptase) in the sub-epithelial connective tissue (Figure 2A). In lung specimens, several and spread immunopositive mast cells surrounded by a positive halo (degranulated tryptase) in both the perivascular interstitium and interalveolar septa were detected; also detectable were areas with spread immunopositivity (Figure 2B). Several immunopositive mast cells surrounded by a positive halo, due to the tryptase degranulation, were highlighted in the splenic tissue (Figure 2C). Finally, scattered immunopositivity of mast cells surrounded by a positive halo, mainly localized in the perivascular areas, was observed in the heart (Figure 3).

Biochemical analyses were performed on blood serum sampled from the iliac vein, revealing increased value of serum tryptase (equal to 94.2 ug/L), troponin I (75,000 pg/mL), and p-BNP (equal to 397.4 pg/mL).

The toxicological investigations were negative for alcohol, abuse substances, and psychotropic drugs.

The cause of death has been assessed as acute myocardial dysfunction due to ventricular fibrillation for myocardial ischemia determined by an allergic coronary vasospasm related to fluorescein hypersensitivity. Therefore, a final diagnosis of type II Kounis syndrome has been delivered.

## 3. Discussion

The presented case describes a rare sudden unexpected death due to an acute myocardial ischemia related to an allergic coronary vasospasm—in a subject with coronary atherosclerosis—mediated by a hypersensitivity reaction to fluorescein, used to perform a retinal fluoroangiography.

Fluoroangiography is an investigation routinely used to study the circulation of both the retina and the choroid through fluorescein, a contrast agent which is injected into the antecubital vein [9]. Similarly to several contrast agents, sodium fluorescein can also lead to adverse events, from mild to severe ones. The rate of such adverse reactions has been reported between 0.083 and 21.69%, with a death rate varying from 1:100,000 to 1:220,000 [10]. Moreover, in the literature, very few fatal cases of myocardial infarction due to fluorescein angiography have been described [11,12].

In the presented case, acute myocardial ischemia related to the hypersensitivity contrast media has caused the death of the man, because of the onset of the so-called Kounis syndrome.

Kounis syndrome is a rare disease, and its real prevalence cannot be certainly established because it is under-recognized and under-reported. Data between 2010 and 2014 from the International Pharmacovigilance Agency reported 51 cases of KS [13]. Akoz et al. [14] described an incidence of KS equal to 27/138.911 among all patients admitted to an emergency department for one year.

To better understand the pathophysiology of Kounis syndrome, a classification system has been provided, identifying three different types [15]. Type I is also known as vasospastic allergic angina or myocardial infarction with non-obstructive coronary arteries (MINOCA). It occurs in subjects with no coronary atherosclerosis in which the anaphylactic inflammatory mediators are capable of causing coronary spasm. Type II involves subjects suffering from coronary atherosclerosis, in which the inflammatory mediators can determine coronary spasms, until plaque rupture with thrombosis. Type III develops in patients with coronary stents, in which the inflammatory molecules promote stent thrombosis. However, even if each type has different pathophysiology, in clinical practice, individual cases have not fit into one specific category because of an overlap between these three types.

Thus, the pathophysiology of Kounis syndrome depends on a complex mechanism based on the interaction between the coronary system and allergic inflammatory mediators, which can determine coronary vasospasm and platelet activation and aggregation. It is well known that some allergic mediators (i.e., tryptase, histamine, chymase) have vasoactive effects (also on heart circulation), being capable of stimulating the coagulation cascade (promoting thrombosis) and leading to the destabilization of atherosclerotic plaques [16,17,18].

Similarly to anaphylaxis, post mortem diagnosis of Kounis syndrome can be difficult, but an implementation of the routinary investigations useful for the assessment of allergic reaction might provide very important information supporting the Kounis syndrome occurrence.

In anaphylactic death, the main gross findings are usually described at the respiratory system. These are usually represented by mucus hypersecretion and mucosal edema at the upper airway, leading to lumen obstruction, strongly supporting hypersensitivity-related death. Massive lung edema can also be found [19,20]. Such macroscopic evidence are also confirmed by routine histological examinations that have also highlighted signs of bronchospasm and, above all, the presence of inflammatory infiltrates mostly composed of eosinophils and mast cells [19,21].

When Kounis syndrome is suspected, particular attention should also be given to the heart and coronary arteries. The microscopic analysis of coronary arteries can reveal the presence of inflammatory infiltrates (i.e., mast cells and eosinophils) in wall vessels, especially in the region of vasospasm occurrence. If coronary arteries are affected by atherosclerotic processes, the inflammatory infiltrates are usually described in the plaques themselves [22,23]. At routine histology, the myocardial tissue might reveal clear signs of myocardial infarction, usually described as foci of coagulative necrosis with neutrophil infiltration [24]. These may not be found in deaths occurring within a short time interval from the onset of ischemia (the so-called early myocardial ischemia); in these cases, signs of myocardial damage, such as myofiber eosinophilia, elongation of sarcomeres and nuclei, wavy fibers, interstitial oedema, and contraction band necrosis can also be observed [25].

In the presented case, both macroscopic and microscopic findings at the upper and lower airways did not support the occurrence of an anaphylactic asphyxia. On the other hand, in the myocardial tissue signs of both acute ischemia (foci of contraction band necrosis) and malignant tachyarrhythmia (myofiber break-up: stretching and/or rupture of myocytes resulting in myocellular segmentation at the intercalated discs associated with changes of myocardial bundles and hypercontracted myocells alternated with hyperdistended ones) were detected [26].

However, in both the respiratory and cardiac tissues the detected findings could not be considered specific and/or effective in certifying a post mortem diagnosis of KS. Therefore, other analyses have been performed. In particular, both immunohistochemistry and biochemistry have been proved to be very useful [8].

Immunohistochemical analysis allows the identification of those immune cells contributing to the inflammatory infiltrates. For this purpose, immunohistochemistry should be performed on anaphylaxis target tissues such as in the respiratory system (i.e., larynx, bronchi, lungs), intestinal mucosa, spleen, and skin [27]. Eosinophils and basophils can be detected using major basic protein (MBP) and pro major basic protein 1 (proMBP1) antibodies [28], respectively. Moreover, the detection of activated mast cells is reported as a basic requirement for considering the occurrence of the hypersensitivity reaction [19]. Mast cells are involved in several physiological processes, such as angiogenesis and tissue remodeling, and non-anaphylactic deaths. Thus, to support the occurrence of anaphylaxis, several studies have been reported in the literature, proposing an analysis of different immunohistochemical markers for the activated mast cell detection. Among these suggested markers, both the anti-stem cell factor (which detects the mature forms of mast cells) and anti-CD117 (identifying tissue mast cells) are considered to be useful [8,19].

The detection of tryptase is also described as an effective tool, given the well-known role of tryptase in the anaphylactic immune cascade. In post mortem settings, anti-tryptase antibody immunopositivity can highlight the presence of both activated mast cells and degranulated tryptase, which strongly support the anaphylactic reaction [19,29]. In fact, forensic literature agrees in considering the detection of abundant degranulated tryptase (together with activated mast cells, using anti-tryptase antibody) as a suggestive marker of anaphylaxis [19].

Another immunohistochemical marker proven to be useful for mast cell detection is chymase, which has been described mostly in mast cells localized in perivascular space [30].

The immunohistochemical detection of tryptase could play a pivotal role also in cases of Kounis syndrome. In fact, it has been suggested that in Kounis syndrome, the related myocardial damage depends on the affection of the cardiovascular system mediated by both mast cell degranulation and inflammatory mediator release (i.e., histamine). Consequently, the detection in coronary arteries and myocardial tissue of both activated and degranulated (tryptase) mast cells should be implemented to demonstrate the allergic involvement of the heart [31].

In the presented case, the usefulness of the immunohistochemical analysis has been confirmed, as the tryptase immunopositivity demonstrated the activation of mast cells with tryptase degranulation in each analyzed tissue. In particular, an important involvement of the cardiac tissue was found, leading to the diagnosis of Kounis syndrome.

Post mortem assessment of anaphylaxis also makes use of the analysis of serum tryptase, measured on peripheral blood sample, as an indicator of systemic mast cell mediator release [32]. The analysis consists predominantly in total tryptase measurement, but some researchers have also tested beta-tryptase, which has been described as more specific for anaphylaxis [32,33]; however, there are no studies comparing these two analyses, such as to prove a greater post mortem utility of beta-tryptase. The main literature evidence has reported femoral or external iliac veins as the most recommended sites for blood sampling [34]. Several tryptase-level cut-offs have been reported in forensic literature. Kounis et al. [22] described a value greater than 10 μg/L as a sensible and specific cut-off, while other researchers proposed a larger cut-off of 45 μg/L for serum tryptase measured on femoral blood [22,35].

Thus, post mortem biochemical investigation should be integrated with analysis of myocardial damage markers. In a clinical setting, just as for acute coronary syndromes, for the diagnosis of Kounis syndrome the biochemical analysis of myocardial necrosis markers has a pivotal role [36]. Thus, the measurement of troponin in a forensic setting provides useful evidence supporting the diagnosis of Kounis syndrome [8]. Moreover, the cardiac involvement can be evaluated using p-BNP as an indicator of myocardial disfunction [37].

Furthermore, in our case, the value of serum tryptase was over the aforementioned cut-offs, demonstrating the activation of those immune cells responsible for the anaphylaxis reaction onset. Meanwhile, the high troponin level detected can be considered as evidence supporting the occurrence of myocardial ischemia. Therefore, beyond macroscopic, histological, and immunohistochemical data detected on the cardiac tissue, both troponins and p-BNP values further support the “integrated” diagnosis of Kounis syndrome.

The reported case highlights the diagnostic difficulties faced by clinical and forensic pathologists in the post mortem assessment of Kounis syndrome, which represents a rare cause of sudden cardiac death related to anaphylaxis.

In conclusion, the KS diagnosis may be performed by the integration of:

- anamnestic data (i.e., previous signs and symptoms of allergic reaction);
- circumstantial data referring to death;
- gross and microscopic heart findings describing coronary atherosclerosis and/or signs of myocardial ischemia;
- immunohistochemical evidence of anaphylactic immune cell activation (i.e., mast cells, basophils, eosinophils) with release of specific inflammatory mediators (i.e., tryptase, chymase) in target tissues and, above all, in the coronary artery wall, or coronary atherosclerotic plaques and myocardial tissue;
- biochemical assessment of an increased value of both mediators of anaphylaxis (tryptase) and markers of myocardial damage (i.e., troponin, p-BNP).

In conclusion, the activity of clinical and forensic pathologists plays a pivotal role in the attempt to correctly identify the cause of death, especially in the occurrence of rare “disorders” such as KS, reducing the pool of incorrect diagnoses which would leave these rare syndromes vulnerable to mistreatment.

## Data Availability

All data are reported in the paper.
